# Supplementary figures and images for: Comparison of Transperitoneal and Retroperitoneal Robotic Partial Nephrectomy for Patients with Completely Lower Pole Renal Tumors
Source: J Clin Med. 2023 Jan 16;12(2):722. doi: 10.3390/jcm12020722 (PMC9860733; doi:10.3390/jcm12020722)

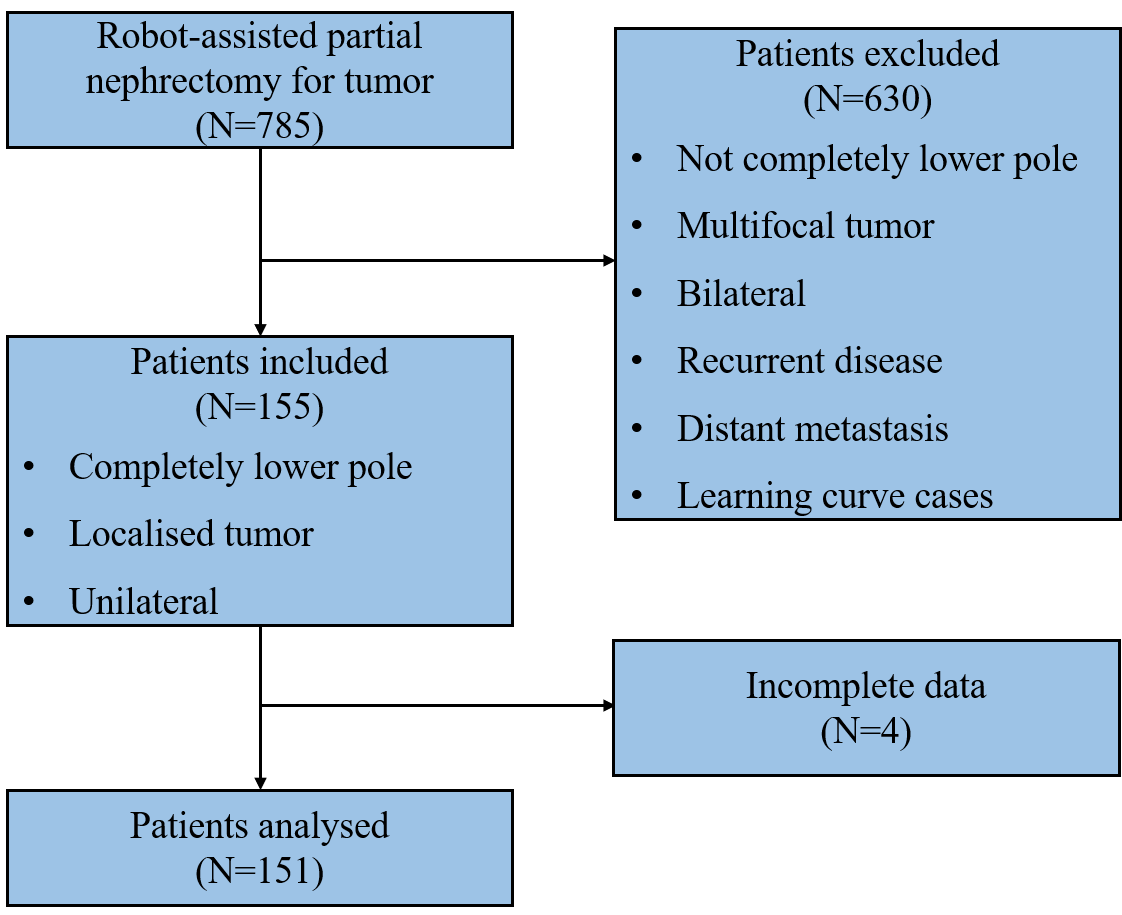

Supplement: Supplementary file 1 [file jcm-12-00722-s001.zip › Figure S1.png]

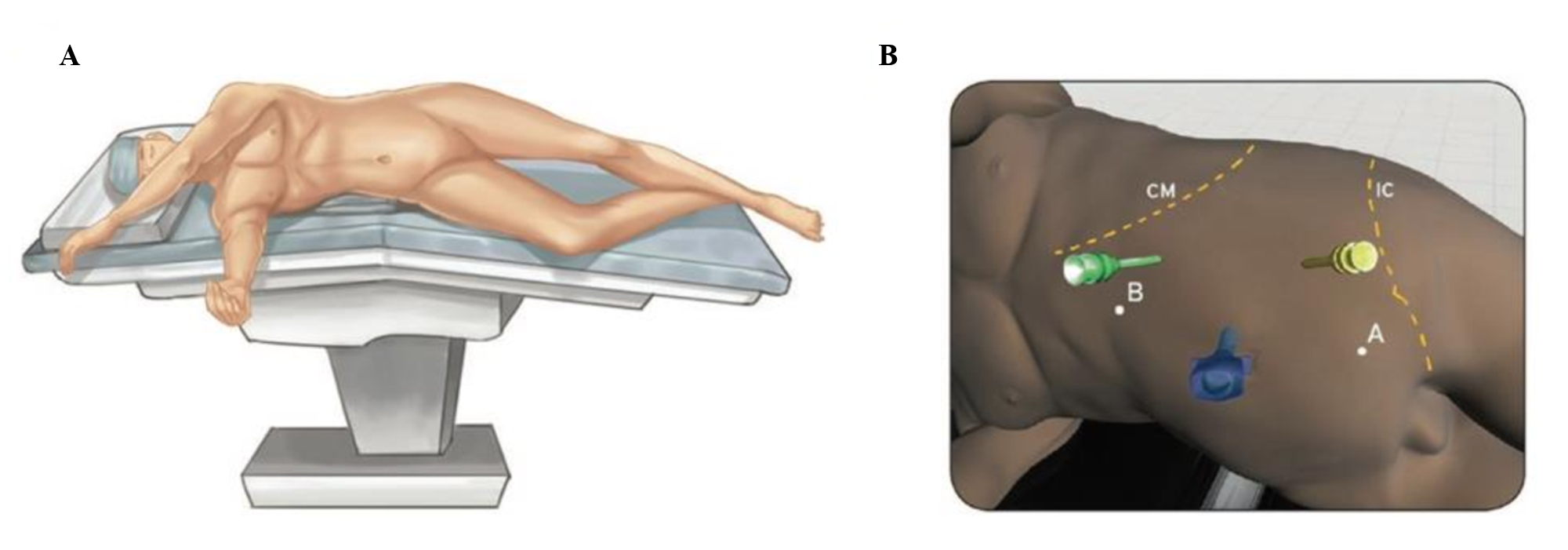

Supplement: Supplementary file 1 [file jcm-12-00722-s001.zip › Figure S2.png]

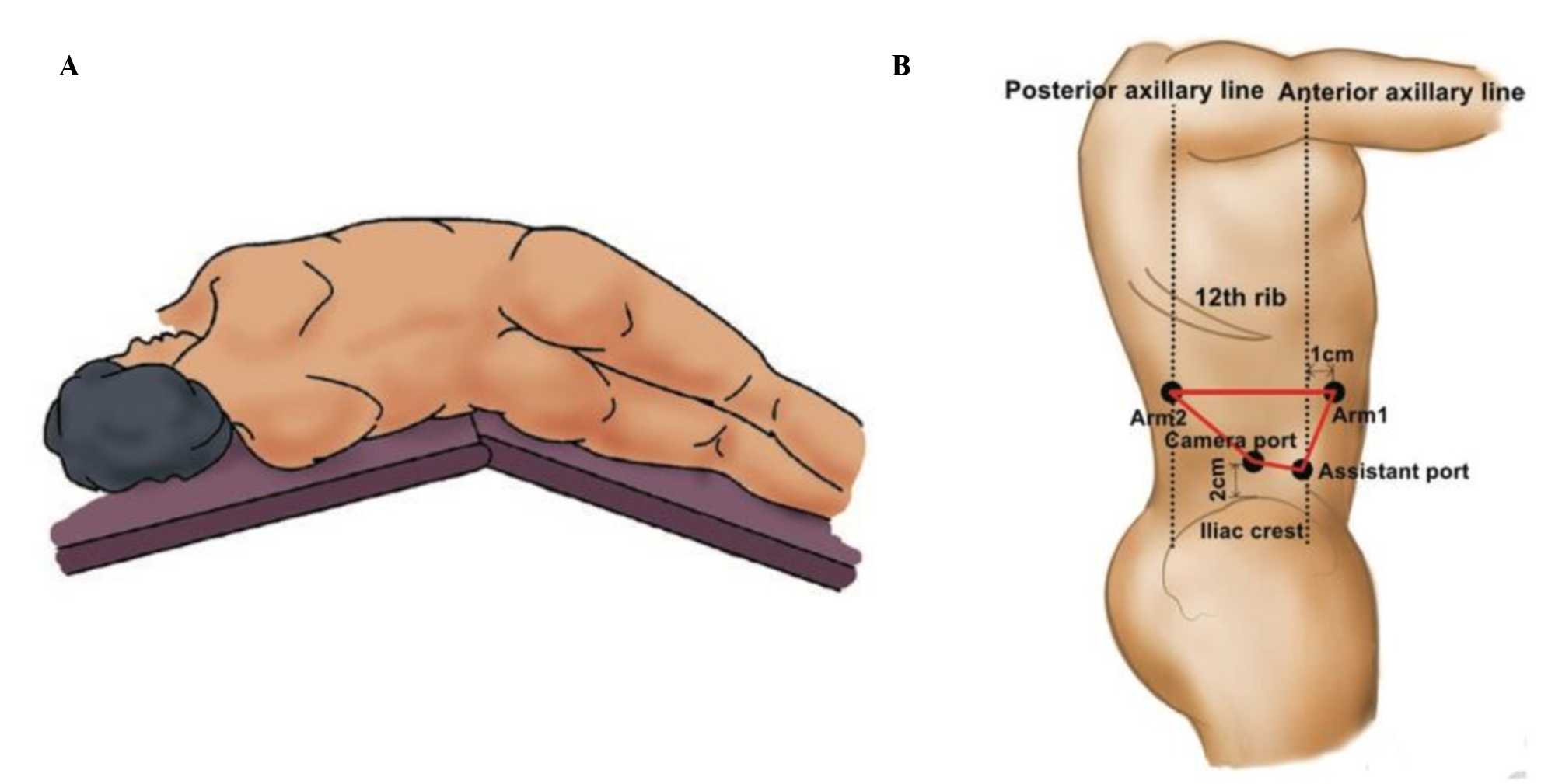

Supplement: Supplementary file 1 [file jcm-12-00722-s001.zip › Figure S3.png]
